# Double-edged sword of technological progress to climate change depends on positioning in global value chains

**DOI:** 10.1093/pnasnexus/pgad288

**Published:** 2023-09-19

**Authors:** Sai Liang, Qiumeng Zhong, Haifeng Zhou, Yihan Liao, Jing You, Jing Meng, Cuiyang Feng, Chen Lin

**Affiliations:** Key Laboratory for City Cluster Environmental Safety and Green Development of the Ministry of Education, School of Ecology, Environment and Resources, Guangdong University of Technology, Guangzhou, Guangdong 510006, P.R. China; Key Laboratory for City Cluster Environmental Safety and Green Development of the Ministry of Education, School of Ecology, Environment and Resources, Guangdong University of Technology, Guangzhou, Guangdong 510006, P.R. China; School of Environment, Beijing Normal University, Beijing 100875, P.R. China; School of Applied Economics, Renmin University of China, Beijing 100872, P.R. China; School of Applied Economics, Renmin University of China, Beijing 100872, P.R. China; The Bartlett School of Sustainable Construction, University College London, London WC1E 6BT, UK; School of Management, China University of Mining & Technology-Beijing, Beijing 100083, P.R. China; School of Applied Economics, Renmin University of China, Beijing 100872, P.R. China

**Keywords:** greenhouse gases, technology, rebound effect, supply chains, climate change

## Abstract

Technological progress (TP) is a double-edged sword to global climate change. This study for the first time reveals rebound and mitigation effects of efficiency-related TP in global value chains (GVCs) on greenhouse gas (GHG) emissions. The integrated effects of TP depend on the positioning of sectors in GVCs. The cost-saving TP in upstream sectors would stimulate downstream demand. This produces stronger rebound effects than mitigation potentials and leads to global GHG emission increments (e.g. TP in the gas sector of China and petroleum and coal products sector of South Korea). In contrast, sectors located in the trailing end of GVCs have greater potentials for GHG emission mitigation through TP, mainly due to the reduction of upstream inputs. (e.g. the construction sector of China and dwelling sector of the United States). Global GHG emissions and production outputs can be either a trade-off or a win–win relationship on account of TP than rebound effects, because TP in different sectors could possibly increase or decrease the emission intensity of GVCs. This study could recognize the most productive spots for GHG emission mitigation through efficiency-related TP. It provides a new perspective for international cooperation to promote global GHG emission mitigation.

**Significance statement**

An interesting and classical question of climate science is how the technological progress affects greenhouse gas (GHG) emissions. By revealing the integrated effects of technological progress on GHG emissions in global value chains (GVCs), we find that the technological progress of upstream manufacturing sectors has stronger rebound effects than mitigation potentials and leads to global GHG emission increments. In contrast, sectors located in the trailing end of GVCs (e.g. the construction sector) have greater potentials for GHG mitigation through technological progress than rebound effects. This study helps recognize the most productive spots for GHG emission mitigation through improving the technologies. Moreover, the newly proposed framework of this study could be applied to other environmental pollutants or resource uses at different scales.

## Introduction

The climate change treaties, such as the Paris Agreement, are expected to promote the joint supply of two global public goods: greenhouse gas (GHG) emission mitigation and knowledge of new technologies that can lower mitigation costs (1). An interesting and classical question is how technological progress (TP) affects GHG emissions. Understanding the impact of TP on global GHG emissions is important for measuring the progress from nationally determined contributions (NDCs) in various technology scenarios (2).

This study mainly focuses on efficiency-related TP. The efficiency-related TP (TP for short in this study) refers to the improvement of the utilization efficiency of inputs due to the invention of new technologies or the improvement of traditional technologies. This is always synonymous with changes in productive capacity at a given level of production factors such as labor and capital (3). Through TP, the same quantity of inputs can produce more products; correspondingly, less inputs are required to produce the same amount of products. For example, TP can save labor by improving the efficiency of machines, innovating organizational systems, or stimulating investment in human capital (e.g. industrial robots reduce labor inputs and innovative organizational structures improve work efficiency) (4); it can save capital by introducing advanced and cheap machines and using more labor-oriented technologies (e.g. intelligent monitoring system reduces maintenance costs) (5); it can also save intermediate inputs through the discovery of new or the improvement of existing technologies that use a smaller proportion of intermediate products (e.g. nanomaterial application improves solar panel efficiency) (6).

The classical understanding of TP's impact is two-fold. On one hand, some studies find that TP can reduce GHG emissions. TP is believed to be the main driving factor of production efficiency improvement and consumption structure transformation (7). Thus, TP is regarded as an effective strategy to reduce GHG emissions (8). On the other hand, lots of studies have discovered that TP can also increase GHG emissions. TP lowers production costs but boosts consumption and outputs (9, 10). As a result, TP leads to an increase in GHG emissions on account of larger production outputs (11). This is regarded as a generalized rebound effect (12). Other studies argue that the relationship between TP and GHG emissions is complicated. TP may increase GHG emissions at the early stage and reduce GHG emissions at the later stage (13). Some regression analyses also show that TP may result in an environmental Kuznets curve (14). It indicates that TP increases GHG emissions when the economic development of a nation is low but begins to reduce GHG emissions when the economic development reaches a certain level (15).

Existing studies have evaluated the impacts of TP on GHG emissions, as well as on energy uses (16) and environmental pollutants (17). They mostly focus on a single sector (18, 19) or a specific economy (20, 21). However, the knowledge of technology is considered as a public goods, not only because of the technology spillover effect (22–24) but also due to the network externality of TP (25, 26). The TP in a nation could generate a global effect on GHG emissions through the global value chains (GVCs) (27). For instance, the TP in the steel sector of China could affect global GHG emissions through the GVCs as follows: Firstly, the efficiency promotion reduces the production costs and direct emission intensity of the steel sector in China (28). Secondly, the reduction in production costs raises the demand for Chinese steel and its complement goods (29). Thirdly, the cost reduction in China lowers the demand for steel products from competitive nations and their GHG emissions (30). Consequently, TP in China makes footprints on global GHG emissions via GVCs (31). However, the cascading effect of TP on global GHG emissions throughout GVCs in a multiregional and multisectoral framework has not been investigated (32). That is, the relationship between the positioning of sectors in GVCs and the integrated impacts of their TP on global GHG emissions throughout GVCs still remains unknown. Revealing this relationship in GVCs is conducive to formulating global GHG emission mitigation policies through TP. It requires a clear description of the internal mechanism to help policymakers see the transparent logic. Some traditional methods only show the quantitative results, leaving the internal mechanism as a black box (33). Thus, the effects of TP in different stages of GVCs on global GHG emissions remain unknown.

Under these backgrounds, this study for the first time reveals the integrated effects of efficiency-related TP in different stages of GVCs on global GHG emissions. It measures the GHG footprint of TP in a newly proposed framework of an environmentally extended general equilibrium model with heterogeneous agent and input–output network (EE-HA-IO), based on the general equilibrium model with heterogeneous agent and input–output network (HA-IO) (34–36). The HA-IO model is related to multisectoral and multiregional models and models with production networks (37–40). It can open the black box of the computable general equilibrium (CGE) models and hence describe the GVCs. The GHG footprint of TP defined in this study refers to the impact of TP in a sector of a nation on GHG emissions from each sector of each nation. The TP of a nation sector means a one-percentage-point increase in its total factor productivity (TFP). Based on this definition, we first provide a method to calculate the GHG footprint of TP using the EE-HA-IO model. Then, we calibrate the EE-HA-IO model with the international input–output database covering 141 nations and 65 sectors from GTAP (version 10) (41). Subsequently, we calculate the GHG footprint of TP in various nations sector and explore the changes in GVCs and associated GHG emissions due to a marginal change in technological levels (i.e. TP).

This study provides a new perspective (i.e. GHG footprint of TP) to evaluate the cascading effects of TP in GVCs on global GHG emissions. It could identify the hotspots of global GHG emission changes due to TP in GVCs under a multiregional and multisectoral framework. The results show that the TP of sectors with different positions in GVCs (measured by the upstreamness of each nation sector) would have different integrated impacts on global GHG emissions. TP in upstream sectors usually leads to global GHG emission increments, while that in downstream sectors usually contributes to global GHG emission reductions. Thus, sector-specific TP policies are required from a GVCs perspective. Furthermore, by applying this framework to specific sectors (i.e. inputting the TFP change caused by actual technology application in a sector into the model), the cascading effect of the actual TP on GHG emissions in GVCs can be revealed. This could provide targeted policy implications for international and intersectoral cooperation to promote global GHG emission mitigation through TP.

## Results

### Critical nations and sectors for the GHG footprint of TP

TP is a double-edged sword that can reduce production costs while boosting consumption and output, which has significant effects on global GHG emissions and climate change. It contributes to the changes of production scale and production efficiency through GVCs and further affects GHG emissions of each nation and sector. TP in the chemical sector of Russia, gas sector of China, and petroleum and coal products sectors of China, the Unite States, and South Korea contributed significantly to global GHG emission increments (Table S1). In contrast, TP in sugar and construction sectors of China and dwelling and human health and social work activities sectors of the United States would promote GHG emission reductions (Table S2).

At the national scale, TP in nations considered in this study would lead to GHG emission increments. This indicates that the rebound effect of TP in sectors is greater than the reduction effect. In particular, TP in China, Russia, and the United States primarily contributes to global GHG emission increments, mainly occurring domestically (occupying 78–95%) (Fig. S1). These nations possess superior technological capabilities, abundant resource reserves, and extensive domestic markets, leading to relatively complete domestic supply chains and consequent domestic effects of TP.

Meanwhile, there are obvious cross-national effects of TP on global GHG emissions (e.g. South Korea, Canada, and Australia). TP in South Korea would lead to an increase in global GHG emissions (628 MtCO_2_-eq, million tonnes of CO_2_ equivalents), where 418 MtCO_2_-eq are embodied in international trade (accounting for 65%). With poor natural resource endowment and relatively small domestic market, South Korea highly depends on international trade. Its exports accounted for 42% of its gross domestic product (GDP) in 2021, significantly exceeding the global average level (29%) (42). Meanwhile, over 90% of its consumed energy and natural resources depend on imports (43). Consequently, TP in South Korea would significantly affect the production and GHG emissions of foreign nations. TP in South Korea would contribute relatively less to its absolute domestic GHG emissions than to foreign nations. However, in terms of GHG emission changing rates, it could lead to a 40% increase in domestic GHG emissions, mainly attributable to GHG emission increments in the electricity sector (Fig. S2).

In addition, we find a significant negative correlation (−0.483, *P* = 0.068) between the GHG footprint of TP of nations and their economic development levels (represented by per capita GDP) (Fig. S3). This means that the GHG footprint of TP would become smaller as the economic level increases. It largely benefits from the life cycle management of technologies and products in developed nations. For example, the United States Environmental Protection Agency and the European Environment Agency have been promoting the life cycle assessment in product design (44, 45). Thus, promoting economic development and life cycle management could help global GHG emission reductions through TP. On the contrary, TP's cross-national impacts on GHG emissions are more pronounced in nations with higher levels of economic development (e.g. South Korea). By conducting a correlation analysis between the proportion of the foreign GHG footprint of TP (i.e. foreign GHG emissions caused by TP in a nation) in the total GHG footprint of TP in a nation and its economic development level, we find a significant positive correlation (0.485, *P* = 0.067). This indicates that TP in nations with higher levels of economic development would make more footprints on GHG emission of foreign nations. With the process of globalization, most developed nations have shifted the high-emission industries to developing nations. Meanwhile, TP in developed nations may promote supply chain optimization (e.g. TP by saving capital) in favor of domestic GHG emission reductions while contributing to the GHG emissions of foreign nations. This can be validated by the higher carbon leakage of developed nations than developing nations (46, 47).

### International linkages for the GHG footprint of TP

**Fig. 1.**
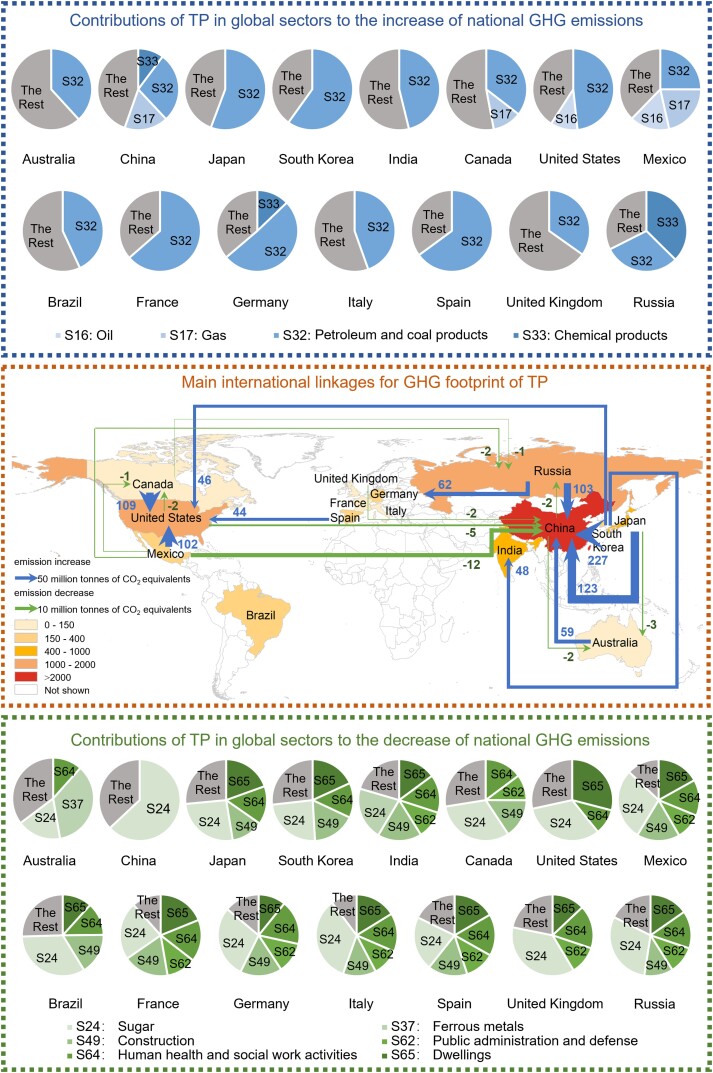
Main international linkages for GHG footprint of TP. The map shading indicates GHG emission changes of 15 major nations due to global TP. The arrows show the 10 largest increase or decrease changes of the GHG emissions affected by TP. The pie charts denote the contributions of TP in global sectors to the increase or decrease of national GHG emissions.

We further explore the cross-national impact of TP on GHG emissions through GVCs. Figure 1 shows the impact of TP in a nation on GHG emissions of other nations. The TP in South Korea could contribute 227 MtCO_2_-eq of GHG emission increments in China (accounting for 2% of China's GHG emissions). The TP in Japan would contribute 123 MtCO_2_-eq of GHG emission increments in China, accounting for 56% of the foreign GHG emission increments resulting from the TP in Japan. In addition, Canada and Mexico both have frequent trade with the United States, and the TP in these nations would contribute 109 and 102 MtCO_2_-eq of GHG emission increments in the United States, respectively.

By further exploring the impact of TP on GHG emissions at the sector level, we find that the upstream and downstream effects are two channels that TP affects GHG emissions in GVCs. On one hand, TP in a sector could contribute to GHG emission growth in its downstream sectors. This is because TP in upstream sectors would reduce their costs and promote those sectors' and downstream sectors' production scale (29). As shown in Fig. 1 (the upper part), TP in the petroleum and coal products sector would contribute significantly to GHG emission growth in all nations. For instance, the combination of horizontal drilling and hydraulic fracturing has increased the production of crude oil in the United States (48), and the energy use has subsequently resulted in significant GHG emissions. In particular, the petroleum and coal products sector in South Korea is the main sector affecting GHG emission growth in China. It contributes 137 MtCO_2_-eq, accounting for 1% of China's GHG emissions (Fig. S4). In particular, TP in this sector contributes 40, 32, and 13 MtCO_2_-eq of GHG emission increments in the chemical products, mineral products, and electricity sectors of China. They account for 5, 2, and 0.3% of GHG emissions from the chemical products, mineral products, and electricity sectors of China, respectively. This is mainly because South Korea produces intermediate inputs for China's manufacturing sectors and China has a long value chain on the downstream side of South Korea (49). TP of the upstream sectors in South Korea promotes the production scale expansion of these downstream sectors in China and leads to higher GHG emissions.

On the other hand, TP in a nation could contribute to GHG emission mitigation in its upstream nations. This is because TP in downstream sectors leads to efficiency gains and reduces the demand for upstream inputs. For example, TP in the United States could reduce 5 MtCO_2_-eq of GHG emissions in China (accounting for 0.04% of China's GHG emissions) and 2 MtCO_2_-eq of GHG emissions in Canada (accounting for 0.4% of Canada's GHG emissions) (Fig. 1). For China, electricity (−4 Mt CO_2_-eq, accounting for 0.1% of GHG emissions from the electricity sector of China) is the main sector where the GHG emission mitigation benefits from TP in the United States (Fig. S5A). TP in the construction sector of the United States could help reduce 2 MtCO_2_-eq of GHG emissions from the electricity sector of China (accounting for 0.04% of GHG emissions from the electricity sector of China). For Canada, the oil and gas sectors (both 7 MtCO_2_-eq, accounting for 1% of GHG emissions of Canada) are main sectors of GHG emission mitigation (Fig. S5B). TP in the petroleum and coal products sector in the United States could help reduce 6 MtCO_2_-eq and 5 MtCO_2_-eq of GHG emissions from the oil and gas sectors of Canada, respectively. They account for 7 and 5% of GHG emissions from the oil and gas sectors of Canada. This is mainly because the final products produced in the United States have a long value chain on its upstream side, which includes energy products from Canada and steel products from China (50, 51). Its TP would reduce inputs from the upstream sectors and leads to lower GHG emissions for unitary product output.

### International linkages for GHG emission intensity changes affected by TP

TP affects global GHG emissions by changing the production scale and GHG emission intensity. We explore the relationship between GHG footprints and outputs of TP in sectors, and the result shows a significantly positive correlation (0.445, *P* = 0.000) when the GHG emission intensity is constant. It generally leads to trade-offs between production increments and GHG emission mitigation. However, this study finds that TP in some food sectors could promote a win–win situation. For example, TP in sugar sectors with relatively downstream positions in GVCs would increase sugar outputs and contribute to global GHG emission reductions (Fig. S6). To balance these two goals and realize win–win situations, the reduction of GHG emission intensity is of vital importance. Therefore, we further investigate the effects of TP on GHG emission intensity of each nation and take it as a critical perspective to explore the GHG footprint of TP.

**Fig. 2.**
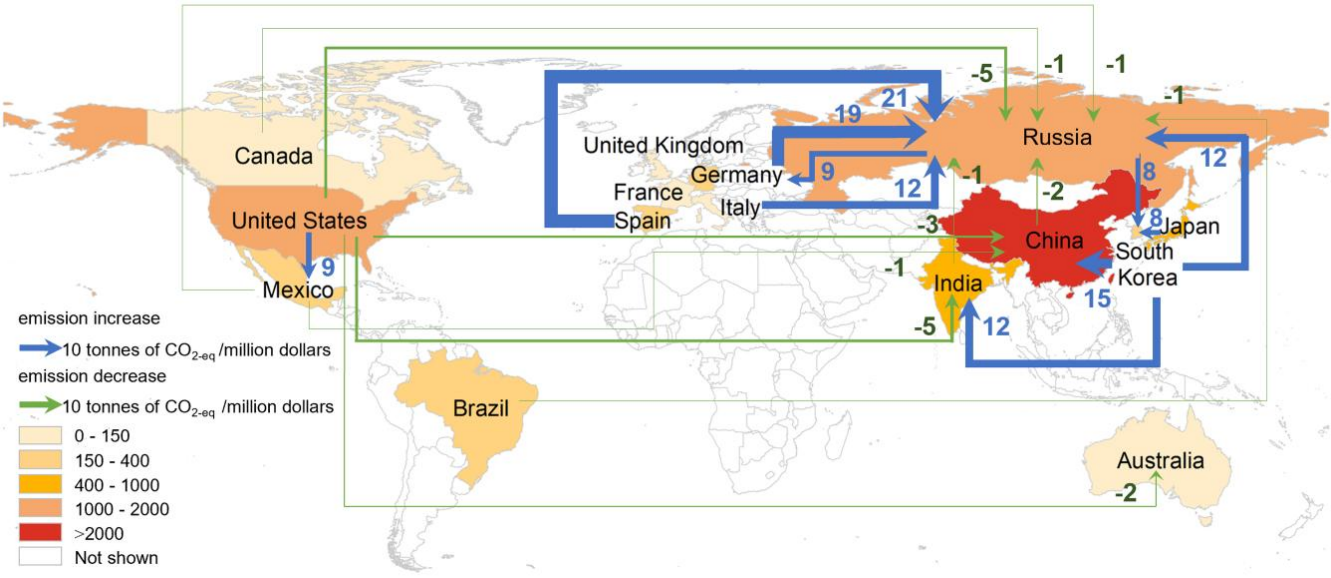
Main international linkages for GHG emission intensity changes affected by TP. The map shading indicates GHG emission changes of 15 major nations due to global TP. The arrows show the 10 largest increase or decrease changes of GHG emission intensity affected by TP.

For a nation, TP could generate a global effect on GHG emission intensity through GVCs (Fig. 2). In a general equilibrium economy, TP affects the emission intensity through two channels. Firstly, TP in a sector could reduce the inputs per unitary production output. Consequently, it decreases direct GHG emission intensity. Secondly, TP could change the sectoral sale structure. Given that emission intensities vary across sectors, the change of sectoral sale structure would change the national emission intensity. For example, TP in the United Kingdom could reduce the GHG emission intensity of China by 0.4 tCO_2_-eq/Md (tonnes of CO_2_ equivalents/million dollars). On one hand, TP in the United Kingdom leads to significant sale increases in China's sectors where GHG emission intensity is below national level (e.g. the construction and machinery and equipment sectors). The sale increases of the construction and machinery and equipment sectors of China mainly result from TP in the upstream metal sectors of the United Kingdom (Table S3). On the other hand, TP in the United Kingdom leads to certain sale decreases in China's sectors where GHG emission intensity is above national level (e.g. the electricity sector). The sale decrease in the electricity sector of China mainly roots in TP in downstream sectors of United Kingdom (e.g. the construction and dwelling sectors). As a result, the sectoral sale structure of China becomes more environmentally friendly because of the TP in the United Kingdom.

Figure 2 also shows that TP in South Korea could increase the GHG emission intensity of China by 15 tCO_2_-eq/Md. On one hand, TP in South Korea leads to significant output increases in China's sectors where GHG emission intensity is above national level (e.g. the petroleum and coal products and chemical products sectors). The sale increases in these sectors mainly result from the TP in upstream or competitive sectors of South Korea (e.g. the gas, petroleum and coal product, and chemical product sectors) (Table S4). On the other hand, TP in South Korea leads to sale decreases in China's certain sectors where GHG emission intensity is below national level, such as the trade and financial service sectors. The sale decreases in these sectors mainly result from the upstream petroleum and coal products sector of South Korea. As a result, the sectoral sale structure of China becomes more pollutional because of the TP in South Korea. This leads to an increase in the national GHG emission intensity of China.

### GHG footprint of TP in gas and construction sectors

To illustrate the rebound or mitigation effects of TP in different stages of GVCs on GHG emissions, we select gas and construction sectors as the representatives of upstream and downstream sectors, respectively. TP in gas sectors would lead to 783 MtCO_2_-eq of global GHG emission increments, and TP in construction sectors would contribute 34 MtCO_2_-eq of global GHG emission reductions.

**Fig. 3.**
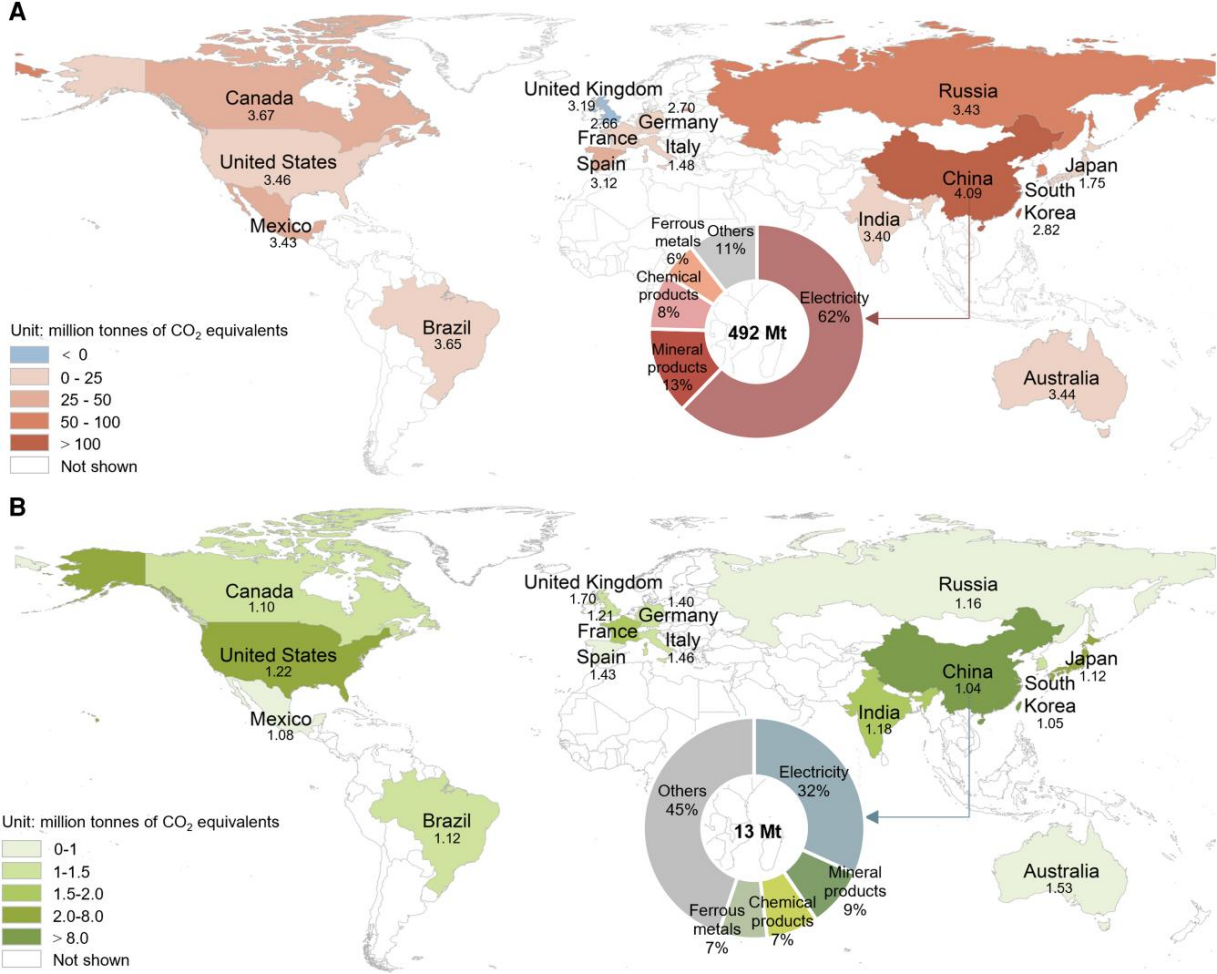
GHG footprint of TP in gas and construction sectors. A) GHG footprint of TP in gas sectors and sectoral structure for China's gas sector. B) GHG footprint of TP in construction sectors and sectoral structure for China's construction sector. The number below or above the name of each nation represents the upstreamness of gas and construction sectors.

Significant TP has been made in natural gas extraction, storage, and distribution. For example, the shale gas revolution would significantly reduce the cost of natural gas production (52), mainly due to the advances in the cost-effectiveness of horizontal drilling, new mapping tools, and hydraulic fracturing technologies (53). By saving capital, TP in the gas sector would stimulate the expansion of downstream sectors and increase energy use and consequent GHG emissions. Figure 3A shows the GHG footprint of TP in the gas sector of nations. TP in the gas sector of China is the most critical by contributing 492 MtCO_2_-eq of global GHG emission increments, most of which occur domestically (468 MtCO_2_-eq, accounting for 95%). The electricity sector is a downstream sector that is strongly connected with the gas sector. TP in gas sector of China would contribute 306 MtCO_2_-eq of GHG emission increments in global electricity sectors (occupying 64% of the total GHG footprint of TP in the gas sector of China), 299 MtCO_2_-eq of which occur domestically.

Although the construction sector is usually regarded as an environmentally friendly sector with low direct GHG emissions, it drives significant indirect emissions from upstream sectors in GVCs (54). Fortunately, we find that the TP in construction sectors could contribute to global GHG emission reductions (Fig. 3B). In particular, TP in China's construction sector would contribute 13 MtCO_2_-eq of global GHG emission reductions (accounting for 37% of the total emission reductions due to TP in global construction sectors). The global electricity sector is the primary beneficiary with a 4 MtCO_2_-eq reduction in GHG emissions (occupying 32% of GHG emission reductions caused by TP in China's construction sector). Construction is a highly energy-consuming sector, with electricity being one of its primary sources. TP in the construction sector could reduce electricity demand by increasing energy use efficiency (e.g. promoting energy-efficient construction design, implementing precise energy management and control, and utilizing renewable energy) (55), thus reducing GHG emissions from the electricity sector. This reflects enormous potentials of TP in downstream sectors to promote emission reductions in upstream sectors.

### Positioning of nation sectors in GVCs affects the GHG footprint of TP

TP in different nation sectors could have positive or negative effects on global GHG emissions. As we discussed previously, TP in a nation sector could contribute to GHG emission growth in its downstream nation sectors and GHG emission mitigation in its upstream nation sectors. In this section, we empirically illustrate the relationship between the positions of nation sectors in GVCs and the GHG footprint of their TP.

This study uses the upstreamness (56) of each nation sector to quantify its position in GVCs. Table 1 shows the relationship between the GHG footprint of TP and upstreamness. The results show a significantly positive correlation between the GHG footprint of TP and the upstreamness. On one hand, TP of a nation sector with relatively upstream position in GVCs (e.g. upstream manufacturing sectors) tends to induce higher global GHG emission increments. Simply depending on TP in these sectors is insufficient for GHG emission mitigation. More effective measures for GHG emission mitigation are urgently needed to complement their TP. For example, the cost-saving technology in the coal mining sector would reduce the price of coal and result in an increase of coal demand (57). Replacing coal by low-cost renewables, which reduces the GHG emission intensity of energy supply sectors, would mitigate GHG emissions. On the other hand, TP in a nation sector with a relatively downstream position in GVCs results in higher global GHG emission reductions. This implies that TP in downstream sectors with long value chains is an effective measure to reduce GHG emissions. Thus, technological investments in downstream sectors can better achieve win–win situations for economic welfare growth and GHG emission reduction.

**Table 1.** Pearson correlation coefficients for GHG footprint of TP and the upstreamness of nation sectors in GVCs.

Selected regions	Pearson correlation coefficients	*P*-values
World	0.029	0.005***
Canada	0.514	0.000***
Australia	0.485	0.000***
Mexico	0.485	0.000***
United Kingdom	0.469	0.001***
China	0.391	0.001***
Brazil	0.304	0.014**
South Korea	0.299	0.016**
Japan	0.285	0.022**
Russia	0.255	0.040**
India	0.250	0.045**

Notes: * represents significant at 10% level; ** represents significant at 5% level; and *** represents significant at 1% level. The “World” region in this table includes all the 141 regions of the model.

## Discussion

This study provides a new perspective on global GHG emission mitigation by measuring the GHG footprint of efficiency-related TP. It can reveal the cascading effect of TP on GHG emissions throughout GVCs under a multiregional and multisectoral framework. This is crucial for policy decisions on global GHG emission mitigation and achieving the Paris Agreement goals. Our findings show that TP of sectors with different positions in GVCs would have different integrated impacts (i.e. rebound or mitigation impacts) on global GHG emissions. There is a significantly positive correlation between the GHG footprint of TP in a nation sector and its upstreamness. Thus, sector-specific TP strategies from the GVC perspective are necessary for global GHG emission mitigation.

Existing studies mainly examine the rebound or mitigation effects of TP on carbon emissions in a single region or sector (named direct effects here) (7, 58), which cannot reveal the cascading effects of TP on GHG emissions in GVCs (named indirect effects here). In this study, we set the input of TP as a 1% increase in TFP of a nation sector in the model. In the real world, the cascading effect of actual TP in a nation sector on GHG emissions in GVCs can be quantified by replacing 1% with the TFP change caused by the actual TP in this nation sector. For instance, numerous studies have measured the change in TFP of different sectors in certain regions (i.e. direct effects) (59, 60). Based on the multiregional and multisectoral framework for the GHG footprint of TP proposed in this study, the cascading effect throughout GVCs (i.e. indirect effects) can be quantified with the TFP change of a particular sector from existing studies. Such an in-depth analysis helps avoid the overestimation or underestimation of the effects of TP in a single sector on global GHG emissions, compared to only considering the direct effects of TP on GHG emissions in existing studies. Moreover, investigating the cascading impacts of TP can reveal international and intersectoral carbon leakage, which is not fully characterized by existing studies on TP. Thus, this study could help prevent TP policy application in a specific region from increasing GHG emissions in other regions (i.e. carbon leakage). Furthermore, by identifying the critical linkages between regions and sectors, cooperative mechanisms on TP could be strengthened to promote global GHG emission mitigation.

This study reveals that TP in upstream sectors has stronger rebound effects than mitigation potentials and would lead to global GHG emission increments (e.g. the gas and petroleum and coal products sectors). In a narrow sense, the rebound effect refers to an increase in energy use induced by an improvement of energy efficiency (61). This study observes that an improvement in TFP can also lead to increases in energy use and GHG emissions. We define this phenomenon as the generalized rebound effect. Moreover, we find that the TP in upstream sectors (e.g. gas sector) would contribute to its own GHG emission increments as well as that of its downstream sectors. The Intergovernmental Panel on Climate Change (IPCC) and World Bank have proposed many programs to facilitate technology development and technology transfer. Several programs focus on the technologies for improving energy exploitation efficiency, such as the Energy and Mineral Sectors Strengthening Project II (62) and Extractive Industries Technical Advisory Facility (63). These programs highlight the importance of TP in upstream sectors. TP in such upstream sectors boosts consumption and output increments in downstream sectors, contributing to global GHG emission increments. This pattern may continue in the context of global economic development. Thus, to promote GHG emission reductions in GVCs, upstream sectors should conduct specific analyses of different technologies. By applying the framework proposed in this study, the sectors could choose the technologies with mitigation or relatively small rebound effects.

On the contrary, we find that the TP in sectors at the trailing end of GVCs has greater potentials of GHG emission mitigation than rebound effects, such as construction and dwelling sectors. TP in these downstream sectors could improve the utilization efficiency for products from upstream value chains, consequently reducing global GHG emissions. Existing programs have highlighted the importance of TP in downstream sectors as consumers in GVCs (64, 65). Further efforts are required to promote TP in downstream sectors for global GHG emission mitigation. For instance, the Chinese government has taken measures to promote TP in the construction sector through policy guidance and standard setting (66). This could help increase the production efficiency and reduce upstream sectoral inputs. In addition, the government could offer incentive rewards to encourage technological innovation in enterprises of downstream sectors. Meanwhile, the government could promote technological cooperation and information sharing among enterprises, thereby promoting the application of new technologies within downstream sectors.

Furthermore, our findings highlight the importance of promoting GHG emission mitigation throughout GVCs from a multiregional and multisectoral framework. It is essential to establish cooperation mechanisms between upstream and downstream sectors. In particular, the cross-sectoral impact of TP on GHG emissions is prominent within a nation. Thus, conducting cross-regional and cross-sectoral management domestically is necessary and feasible to promote GHG emission reductions. The government could develop a joint GHG regulatory mechanism for closely linked sectors due to the cascading effects of TP (e.g. gas–electricity and construction–electricity sector pairs in China). This joint mechanism allows upstream and downstream sectors of critical supply chains to share the responsibility of GHG emission control from the TP perspective, thus promoting a joint effort in supply chains to reduce global GHG emissions. In particular, the development of technologies and transfer of technologies to downstream sectors should be significantly encouraged.

In the context of growing international trade, this study reveals the cross-national effects of TP on GHG emissions. Existing studies from the consumption perspective have highlighted the flows of embodied carbon emissions between the United States and developing economies (e.g. China) (67). This study has newly identified critical international pairs from the TP perspective (e.g. South Korea–China). TP in South Korea would significantly contribute to GHG emission increments in foreign nations (e.g. China). Such nations should take more responsibility of GHG emission mitigation from a TP perspective as they reap economic and environmental benefits through GVCs. It is necessary to promote the cooperation between South Korea and China in terms of technologies and management. Taking advantage of South Korea's experiences and technologies to promote TP in downstream sectors of China can reduce GHG emissions in GVCs. Conversely, TP in some nations has positive effects on GHG emission reductions in other nations. For example, TP in the United States could contribute to GHG emission mitigation in China and Canada. These nations should strengthen their cooperation on technological innovation and jointly promote technological development and applications (especially in downstream sectors).

In addition, comparing with the developed nations, TP in developing nations is rather slow. More attention is paid to improving the living standards in less-developed nations, and thus, TP gets less inputs (68). Spurring the innovation and development of technologies in developing nations (especially their downstream sectors) is also a promising method to achieve GHG emission mitigation. Thus, developed nations are supposed to help developing nations promote TP, such as increasing technological investment and accelerating technology transfer (especially for the downstream sectors). Policy coordination and information sharing can be enhanced through international climate change cooperation mechanisms to promote global GHG emission mitigation.

Uncertainties of the results mainly come from the substitution elasticity of intermediate inputs θ in production functions and the global multiregional input–output (MRIO) data. The change of parameter θ might have an effect on the substitution relationship between products and price formation mechanism. To test the robustness of results, this study investigates three possible values of the parameter θ (θ∈{0.4,0.6,0.9}) according to Baqaee and Farhi (36). Robustness checks show that our results are not sensitive to the exact value of the parameter **θ**. Under the value volatility of the parameter **θ**, we draw a similar conclusion that the cost-saving TP in upstream sectors has stronger rebound effects while sectors located in the trailing end of GVCs have greater potentials of GHG emission mitigation by TP. In addition, the quality of global MRIO data could lead to uncertainties in the results. This study obtains the global MRIO data from the Global Trade Analysis Project (GTAP) (41), due to its considerably high resolutions of regions and comparable sectors. It gives priority to more reliable data sources by a quality control process. The comparative studies of global MRIO databases reveal that the GTAP database has similar results with WIOD (69) and Eora (70) for most regions. However, standard deviations or other uncertainty information of raw data in the GTAP database is unavailable (71). As a result, it is difficult to do the quantitative uncertainty analysis for the global MRIO data used in this study. If more information on the uncertainty of GTAP data is available, a precise quantitative uncertainty analysis could be conducted in future work.

This study mainly focuses on the efficiency-related TP, and fundamental process innovation of TP (e.g. development of carbon-free technology) is not in the scope of this study. The TFP plays a crucial intermediary role in measuring the economic impact and practical significance of TP (59). In this study, efficiency-related TP is defined as a one-percentage-point increase in the TFP of a sector. Apart from the efficiency, other factors (e.g. carbon cap and innovative technologies) would also affect TP and the associated GHG emissions. The scope of this study is positioned to be the efficiency property of TP. Future studies can incorporate more factors to investigate the impact of generalized TP on GHG emissions. This requires the development of more sophisticated models. In addition, this study takes 2014 as the base year given the data availability. Future work could update the results to more recent time points, which requires the updating of global MRIO data.

## Materials and methods

**Fig. 4.**
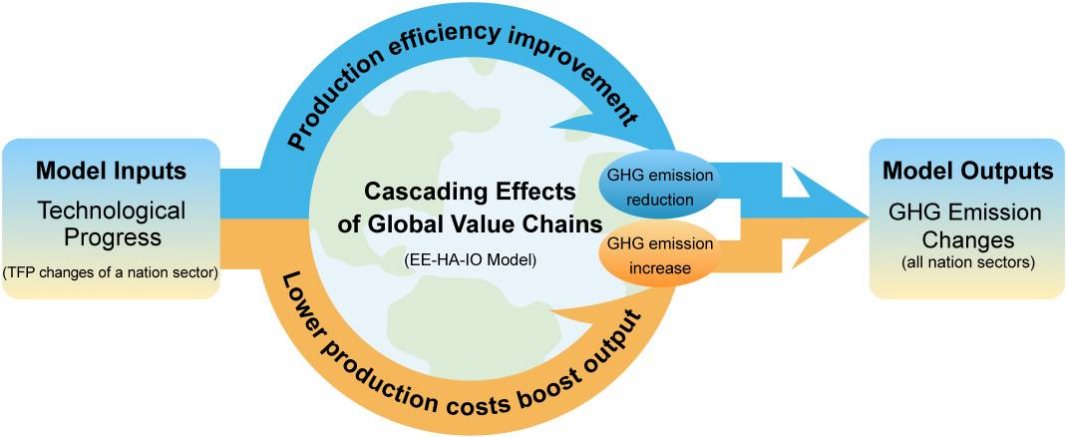
The analytical framework of the EE-HA-IO model.

This study developed an EE-HA-IO to reveal the impact of TP (one-percentage-point increase in the TFP) in a sector of a nation on the changes in global GHG emissions. When the TP happens in a sector of a nation, there are two types of impacts (Fig. 4): production efficiency improvement and lower production costs boosting outputs. These two impacts will be transferred in GVCs and produce cascading effects on global GHG emissions.

### EE-HA-IO

The EE-HA-IO model is based on the HA-IO model of Baqaee and Farhi (36) and is used to evaluate GHG emission changes in different TP scenarios. The EE-HA-IO model treats GHG emissions as the satellite account and constructs a broad class of multisectoral general equilibrium models with heterogeneous agents and intermediate inputs. It unbundles interacting reduced-form building blocks between the representative agent model and the aggregate production function. Here, we use the global MRIO table and sectoral GHG emissions to construct the EE-HA-IO model. On account of the detailed classification of regions and comparable sectors, the global MRIO table is derived from the GTAP (version 10) database (41, 72). GHG emissions in this study include CO_2_, methane (CH_4_), nitrous oxide (N_2_O), and the group of fluorinated gases (F-gas) (Table S5). Sectoral CO_2_ emissions from fossil fuel combustion are obtained from the GTAP-E Data Base (41, 72), and CO_2_ emissions from other sources (i.e. fugitive emissions from fuels, industrial processes and product uses, and wastes) are derived from the Emissions Database for Global Atmospheric Research (EDGAR, version 6.0) database (73) (Table S6). The non-CO_2_ emissions are obtained from Non-CO_2_ Data Base (74). The EE-HA-IO model in this study includes 141 regions and 65 sectors per region.

### Changes in GHG emissions resulting from TP

The EE-HA-IO model includes a set of consumers *C*, producers *N*, factors *F*, and emission *E*. For the economic part of the model (Eqs. (1)–(18)), we keep the same settings with Baqaee and Farhi (36). From Eq. (19), we extend the model to cover GHG emissions. In the multisectoral structure model, each producer uses intermediate inputs and factors to produce one kind of goods, which can be used as intermediate inputs to other producers or final goods to consumers. The emission is a by-product of production processes. Factors are produced in a vacuum. For each agent *c*, the utility maximization activity is shown in Eqs. (1) and (2):


(1) $$\max\;U_{c}(c_{c1},c_{c2},\ldots ,c_{cN}),$$

(2) $$\overset{N}{\underset{\;j=1}{\sum }}\;{\;p}_{j}c_{cj}=\overset{F}{\underset{\;f=1}{\sum }}\;w_{f}\;L_{cf}+\pi_{c},$$

where $U_{c}$ refers to a homothetic utility, $p_{j}$ is the price of goods *j*, $c_{cj}$ is consumer *c*’s consumption of goods *j*, $w_{f}$ is the price of factor *f*, $L_{cf}$ is consumer *c*’s supply of factor *f*, and $\pi_{c}$ is consumer *c*’s share of profits.

The HA-IO matrix is defined to be the (*C* + *N* + *F*) × (*C* + *N* + *F*) matrix **Ω**, as shown in Eq. (3):


(3) $$\Omega =\left(\begin{array}{ccc} O & \Omega_{CN} & O\\ O & \Omega_{NN} & \Omega_{NF}\\ O & O & O \end{array}\right),$$

where Ω indicates the direct inputs from one producer to another producer, which is the analog of the input–coefficient matrix with households endogenous in the context of input–output analysis (IOA), and O refers to the matrix of zeros with proper dimensions.

The *ci*th element of $\Omega_{CN}$ is shown in Eq. (4):


(4) $$\omega_{ci}=\frac{{\;p}_{i}c_{ci}}{\sum_{i=1}^{N}\;{\;p}_{i}c_{ci}},$$

where $\omega_{ci}$ indicates the share of goods *i* in consumer *c*’s expenditures, $p_{i}$ is the price of good *i*, and $c_{ci}$ is consumer *c*’s consumption of goods *i*.

The *ji*th element of $\Omega_{NN}$ is shown in Eq. (5):


(5) $$\omega_{\;ji}=\frac{{\;p}_{i}x_{\;ji}}{s_{j}}=\frac{{\;p}_{i}x_{\;ji}}{{\;p}_{j}y_{j}},$$

where $\omega_{\;ji}$ indicates goods *j*’s expenditures on inputs from *i* as a share of its total revenues and $s_{j}$ is the *j*th element of the sale vector s.

The *jf*th element of $\Omega_{NF}$ is shown in Eq. (6):


(6) $$\omega_{\;jf}=\frac{w_{f}\;L_{\;jf}}{s_{j}}=\frac{w_{f}\;L_{\;jf}}{{\;p}_{j}y_{j}},$$

where $\omega_{\;jf}$ indicates *j*’s expenditures on factor *f* as a share of its total revenues.

The HA-IO Leontief inverse matrix is shown in Eq. (7):


(7) $$\Psi =(I-\Omega {)}^{-1},$$

where Ψ indicates the direct and indirect linkages through the GVCs and I is an identity matrix.

We then define factor–distribution matrix Φ, and the *cf*th element of Φ is shown in Eq. (8):


(8) $$\phi_{cf}=\frac{w_{f}\;L_{cf}}{w_{f}\;L_{f}},$$

where $\phi_{cf}$ indicates the share of factor *f*'s income distributed to consumer *c*.

For the Domar weight, the Domar weight of producer *j* is shown in Eq. (9):


(9) $$\lambda_{j}=\frac{{\;p}_{j}y_{j}}{\mathrm{GDP}},$$

where $\lambda_{j}$ indicates the share of producer *j*’s sales in global GDP.

The Domar weight of factor *f* is shown in Eq. (10):


(10) $$\Lambda_{f}=\frac{w_{f}\;L_{f}}{\mathrm{GDP}},$$

where $\Lambda_{f}$ indicates the share of factor *f*’s value in global GDP.

We decompose the Domar weight of producer *j*, as shown in Eq. (11):


(11) $$\lambda_{i}^{c}=\overset{N}{\underset{\;j=1}{\sum }}\;\omega_{cj}\psi_{\;ji},$$

where $\lambda_{i}^{c}$ indicates the sum of all paths from producer *i* to consumer *c* weighted by that consumer's size and $\psi_{\;ji}$ is the *ji*th element of Ψ.

Then, we decompose the Domar weight of factor *f*, as shown in Eq. (12):


(12) $$\Lambda_{f}^{c}=\overset{N}{\underset{\;j=1}{\sum }}\;\omega_{cj}\psi_{\;jf},$$

where $\Lambda_{f}^{c}$ indicates the sum of all paths from factor *f* to consumer *c* weighted by that consumer's size and $\psi_{\;jf}$ is the *jf*th element of Ψ.

For each producer *k*, the constant elasticity substitution (CES) production function is shown in Eq. (13):


(13) $$\frac{y_{k}}{{\bar{y}}_{k}}=\frac{A_{k}}{{\bar{A}}_{k}}{\left(\overset{F}{\underset{\;f=1}{\sum }}\;\omega_{kf}{\left(\frac{L_{kf}}{{\bar{L}}_{kf}}\right)}^{\frac{\theta_{k}-1}{\theta_{k}}}+\overset{N+F}{\underset{l=1}{\sum }}\;\omega_{kl}{\left(\frac{x_{kl}}{{\bar{x}}_{kl}}\right)}^{\frac{\theta_{k}-1}{\theta_{k}}}\right)}^{\frac{\theta_{k}}{\theta_{k}-1}},$$

where $y_{k}$ is the total output of producer *k*, $A_{k}$ is the technology of producer *k*, and it is a Hicks-neutral shifter, which can be explained as the index of the level of technology. If l∈F, $x_{kl}$ is the input of factor *l* to producer *k* and $\omega_{kl}$ is the element of $\Omega_{NF}$. If l∈N, $x_{kl}$ is the intermediate input of goods of *l* to producer *k* and $\omega_{kl}$ is the element of $\Omega_{NN}$. The notations ${\bar{y}}_{k}$, ${\bar{A}}_{k}$, and ${\bar{x}}_{kl}$ refer to the total output, technology, and inputs of producer *k* in baseline year, respectively. The notation $\theta_{k}$ is the element of θ, indicating the substitution elasticity of inputs for producer *k*.

For the elasticities of sale shares or Domar weights to different productivities, it is shown in Eq. (14):


(14) $$\frac{d\log\;\lambda_{i}}{d\log\;A_{k}}=\underset{j}{\sum }\frac{\lambda_{j}}{\lambda_{i}}(\theta_{j}-1)\mathrm{Co}v_{\Omega_{\left(j\right)}}(\Psi_{\left(k\right)},\Psi_{\left(i\right)})-\underset{g}{\sum }\;\underset{j}{\sum }\frac{\lambda_{j}}{\lambda_{i}}(\theta_{j}-1)\mathrm{Co}v_{\Omega_{\left(j\right)}}(\Psi_{\left(g\right)},\Psi_{\left(i\right)})\frac{d\log\;\Lambda_{g}}{d\log\;A_{k}}+\frac{1}{\lambda_{i}}\underset{g}{\sum }\;\underset{c}{\sum }\;(\lambda_{i}^{c}-\lambda_{i})\phi_{cg}\Lambda_{g}\frac{d\log\;\Lambda_{g}}{d\log\;A_{k}},$$

where $\frac{d\log\;\lambda_{i}}{d\log\;A_{k}}$ indicates the elasticities of sale shares or Domar weights of *i* to the technology of producer *k* and $\mathrm{Co}v_{\Omega_{\left(j\right)}}(\Psi_{\left(k\right)},\Psi_{\left(l\right)})$ is the input–output covariance, as shown in Eq. (15):


(15) $$\mathrm{Co}v_{\Omega^{\left(j\right)}}(\Psi_{\left(k\right)},\Psi_{\left(l\right)})=\underset{i}{\sum }\;\omega_{\;ji}\psi_{ik}\psi_{il}-\left(\underset{i}{\sum }\;\omega_{\;ji}\psi_{ik}\right)\left(\underset{i}{\sum }\;\omega_{\;ji}\psi_{il}\right),$$

where $\Omega^{\left(j\right)}$ is the *j*th row of **Ω** and $\Psi_{\left(k\right)}$ and $\Psi_{\left(l\right)}$ are the *k*th and *l*th columns of Ψ, respectively.

For the elasticities of factor shares to different productivities, it is shown in Eq. (16):


(16) $$\frac{d\log\;\Lambda_{f}}{d\log\;A_{k}}=\underset{j}{\sum }\frac{\lambda_{j}}{\Lambda_{f}}(\theta_{j}-1)\mathrm{Co}v_{\Omega_{\left(j\right)}}(\Psi_{\left(k\right)},\Psi_{\left(f\right)})-\underset{g}{\sum }\;\underset{j}{\sum }\frac{\lambda_{j}}{\Lambda_{f}}(\theta_{j}-1)\mathrm{Co}v_{\Omega_{\left(j\right)}}(\Psi_{\left(g\right)},\Psi_{\left(f\right)})\frac{d\log\;\Lambda_{g}}{d\log\;A_{k}}+\frac{1}{\Lambda_{f}}\underset{g}{\sum }\;\underset{c}{\sum }\;(\Lambda_{f}^{c}-\Lambda_{f})\phi_{cg}\Lambda_{g}\frac{d\log\;\Lambda_{g}}{d\log\;A_{k}},$$

where $\frac{d\log\;\Lambda_{f}}{d\log\;A_{k}}$ indicates the elasticities of factor shares of *f* to the technology of producer *k*.

For the elasticities of wages to different productivities, the calculation is shown in Eq. (17):


(17) $$\frac{d\log\;w_{f}}{d\log\;A_{k}}=\frac{d\log\;\Lambda_{f}}{d\log\;A_{k}}+\frac{d\log\;\gamma }{d\log\;A_{k}}=\frac{d\log\;\Lambda_{f}}{d\log\;A_{k}}+\lambda_{k},$$

where $\frac{d\log\;w_{f}}{d\log\;A_{k}}$ indicates the elasticities of wages of factor *f* to the technology of producer *k* and $\frac{d\log\;\gamma }{d\log\;A_{k}}$ indicates the elasticities of aggregate output γ to the technology of producer *k* and is given by its Domar weight $\lambda_{k}$.

Thus, the elasticities of output quantities of different producers to different productivities are given by Eq. (18):


(18) $$\frac{d\log\;y_{i}}{d\log\;A_{k}}=\frac{d\log\;s_{i}}{d\log\;A_{k}}-\frac{d\log\;{\;p}_{i}}{d\log\;A_{k}}=\left(\frac{d\log\;\lambda_{i}}{d\log\;A_{k}}+\frac{d\log\;\gamma }{d\log\;A_{k}}\right)-\left(-\psi_{ik}+\underset{g}{\sum }\;\psi_{ig}\frac{d\log\;w_{g}}{d\log\;A_{k}}\right)=\frac{d\log\;\lambda_{i}}{d\log\;A_{k}}+\lambda_{k}+\psi_{ik}-\underset{g}{\sum }\;\psi_{ig}\frac{d\log\;w_{g}}{d\log\;A_{k}},$$

where $\frac{d\log\;y_{i}}{d\log\;A_{k}}$ indicates the elasticities of output quantities of producer *i* to the technology of producer *k*.

The economic implication of Eq. (18) is shown in the following, taking TP occurred in sector *k* as an example.

TP and TFP are inextricably linked. The variable of TFP plays a crucial intermediary role in measuring the economic impact and practical significance of TP. TFP refers to the part of the economic growth process that cannot be explained by the number of inputs, while technology is the input-use efficiency that is independent of the number of inputs. Therefore, both the growth of TFP and TP implies an intensive growth mode, representing the “qualitative” part of the economy rather than the “quantitative” one. Moreover, based on assumptions such as constant returns to scale, competitive market, and economic efficiency (75), sectoral TFP just represents TP, and these assumptions are always satisfied when using input–output models as in this study. Thus, the TP in sector *k* (**TP***_k_*) is defined as one-percentage-point increase in the TFP of sector *k*, as shown in Eqs. (19) and (20):


(19) $$TFP_{k}=A_{k},$$

(20) $$TP_{k}=△TFP_{k}=0.01\;TFP_{k}=0.01\;A_{k}.$$

For $\frac{d\log\;s_{i}}{d\log\;A_{k}}$, when the TP occurred in sector *k*, the relative price of products in sector *k* falls. If sector *i* uses product *k* as a production input (i.e. *i* and *k* are complementary goods), then *i* will face lower production cost according to *k*'s relative price changes. As the price of inputs decreases, the output and sales of sector *i* will rise. Finally, because of the complementary relationship between *i* and *k*, the positive technological impact of sector *k* leads to an increase in the total output of sector *i*. This in turn leads to an increase in the emissions generated by the production activities of sector *i*. On the contrary, if sector *k* and sector *i* are in a competitive relationship, the relative price advantage brought by TP of sector *k* will squeeze the market share of sector *i*. The output of sector *i* will decline, which leads to a reduction in emissions generated by sector *i*.

For $\frac{d\log\;{\;p}_{i}}{d\log\;A_{k}}$, when the TP in sector *k* occurs, the TP that occurred in sector *k* will lead to the relative price change of production factor *g*. Sector *i* will adjust factor allocation to respond to the price changes in *g*. If the price of *g* decreases, then sector *i* will increase the use of *g*. Consequently, the output of sector *i* will increase, which leads to more emissions from production activities of sector *i*. If the price of *g* increases, sector *i* will reduce the use of *g*. The output of sector *i* will reduce, and then, emissions of sector *i* will decrease.

In general, the direction of the impact of TP in sector *k* on the output of sector *i* is ambiguous. It is determined by the elasticity of substitution of the two products and their respective elasticity of substitution to the factors.

The GHG emissions from producer *k* are shown in Eq. (21):


(21) $$E_{k}=e_{k}\times \frac{y_{k}}{A_{k}},$$

where $E_{k}$ is the GHG emissions from producer *k*, $e_{k}$ is the GHG emission coefficient of producer *k*, and $\frac{y_{k}}{A_{k}}$ refers to the combination of inputs in the form of CES, which decides the energy usage. Therefore, the elasticity of the GHG emissions from producer *i* to the technology of producer *k* is given by Eq. (22):


(22) $$\frac{d\log\;E_{i}}{d\log\;A_{k}}=\frac{d\log\;y_{i}}{d\log\;A_{k}}-I_{i,k},$$

where $I_{i,k}$ is the *ik*th element of identity matrix **I**. $I_{i,k}=1$ when i=k and $I_{i,k}=0$ when i≠k.

The matrix of GHG emission changes from various producers due to unitary productivity changes in different producers, M, is given by Eq. (23):


(23) $$M=\hat{E}\times \frac{d\log\;E}{d\log\;A},$$

where $\hat{E}$ is the diagonalization of E, which refers to the GHG emissions from various producers in baseline year.

The matrix of GHG emission changes from various producers due to one percent of productivity growth, H, is then calculated by Eq. (24):


(24) $$H=M\times \hat{\Delta A/A},$$

where $\hat{\Delta A/A}$ is the diagonalization of ΔA/A and ΔA/A indicates the growth rate of technology (i.e. 1% in this study).

The matrix of changes in sales from various producers due to one percent of productivity growth of different producers, T, is calculated as follows:


(25) $$T=\hat{s}\times \frac{d\log\;s}{d\log\;A}\times \Delta A/A,$$

where $\hat{s}$ is the diagonalization of s.

The change in GHG emission intensity of producer *i* due to the change in productivity of producer *k*, $D_{i,k}$, is given as follows:


(26) $$D_{i,k}=\frac{E_{i}+H_{i,k}}{s_{i}+T_{i,k}}-\frac{E_{i}}{s_{i}},$$

where $H_{i,k}$ and $T_{i,k}$ are the *ik*th elements of matrixes H and T, respectively.

### Parameter calibration

In this study, the HA-IO matrix **Ω** comes from the global MRIO table derived from the GTAP (version 10) database (41, 72). Specifically, the matrix $\Omega_{CN}$ is calculated based on the final demand matrix of the global MRIO table; the matrix $\Omega_{NN}$ is calculated based on the intermediate input matrix and the total output vector of the global MRIO table; and the matrix $\Omega_{NF}$ is calculated based on the labor vector and the total output vector of the global MRIO table. To eliminate the effects of incorrect data in the global MRIO table, this study conducts parameter calibrations. If an element was smaller than 10% of both the sectoral and national average levels for labor input rates (i.e. the ratio of labor inputs to sales) or larger than 1 in the matrix $\Omega_{NF}$, it will be replaced by the sectoral average level in the subglobal area that its nation belongs to (Table S7). Similar calibrations are done to elements larger than 1 in the matrix $\Omega_{NN}$ (Table S7). In addition, values of the vector θ are from Baqaee and Farhi (36). Other parameters are calculated based on the above parameters.

## Supplementary Material

pgad288_Supplementary_Data

## Data Availability

The data used in the EE-HA-IO model include the global MRIO data in 2014, sectoral GHG emissions of nations, and substitution elasticities of intermediate inputs in production functions. The global MRIO table is derived from GTAP (version 10) database (41, 72). The sectoral GHG emissions of nations are obtained from GTAP-E 10 Data Base (41, 72), non-CO_2_ Data Base (74), and EDGAR (version 6.0) database (73). The substitution elasticities of intermediate inputs in production functions are obtained from Baqaee and Farhi (36).
