# Supplementary material for: Double-edged sword of technological progress to climate change depends on positioning in global value chains
Source: PNAS Nexus. 2023 Sep 19;2(9):pgad288. doi: 10.1093/pnasnexus/pgad288 (PMC10508203; doi:10.1093/pnasnexus/pgad288)
Supplement: pgad288_Supplementary_Data [file pgad288_supplementary_data.zip › PNASNEXUS-PNASNEXUS-2023-00802R-s02.docx]

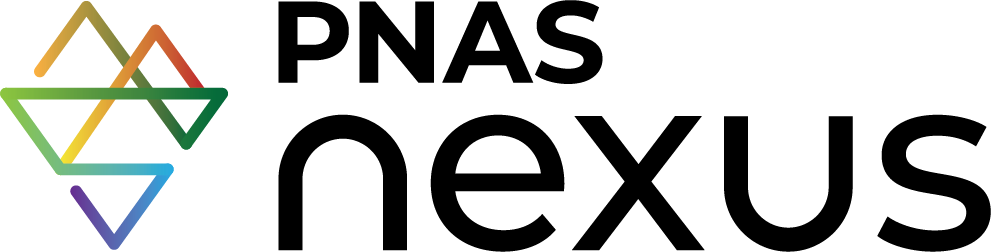


**Supporting Information for**

Double-edged sword of technological progress to climate change depends on positioning in global value chains

Sai Liang^a,#,*^, Qiumeng Zhong^a,#^, Haifeng Zhou^b^, Yihan Liao^c^, Jing You^c^, Jing Meng^d^, Cuiyang Feng^e^, and Chen Lin^c^

^a^ Key Laboratory for City Cluster Environmental Safety and Green Development of the Ministry of Education, School of Ecology, Environment and Resources, Guangdong University of Technology, Guangzhou, 510006, P.R. China

^b^ School of Environment, Beijing Normal University, Beijing 100875, P.R. China

^c^ School of Applied Economics, Renmin University of China, Beijing 100872, P.R. China

^d^ Bartlett School of Sustainable Construction, University College London, London WC1E 7HB, UK

^e^ School of Management, China University of Mining & Technology-Beijing, Beijing, 100083, P.R. China

# These two authors have equal contribution: Sai Liang and Qiumeng Zhong.

* To whom correspondence may be addressed. liangsai@gdut.edu.cn (Sai Liang).

**Figure S1. GHG footprint of technological progress in 15 major nations.**

**Figure S2. The changing rate of GHG emissions due to the technological progress in 15 major nations.**


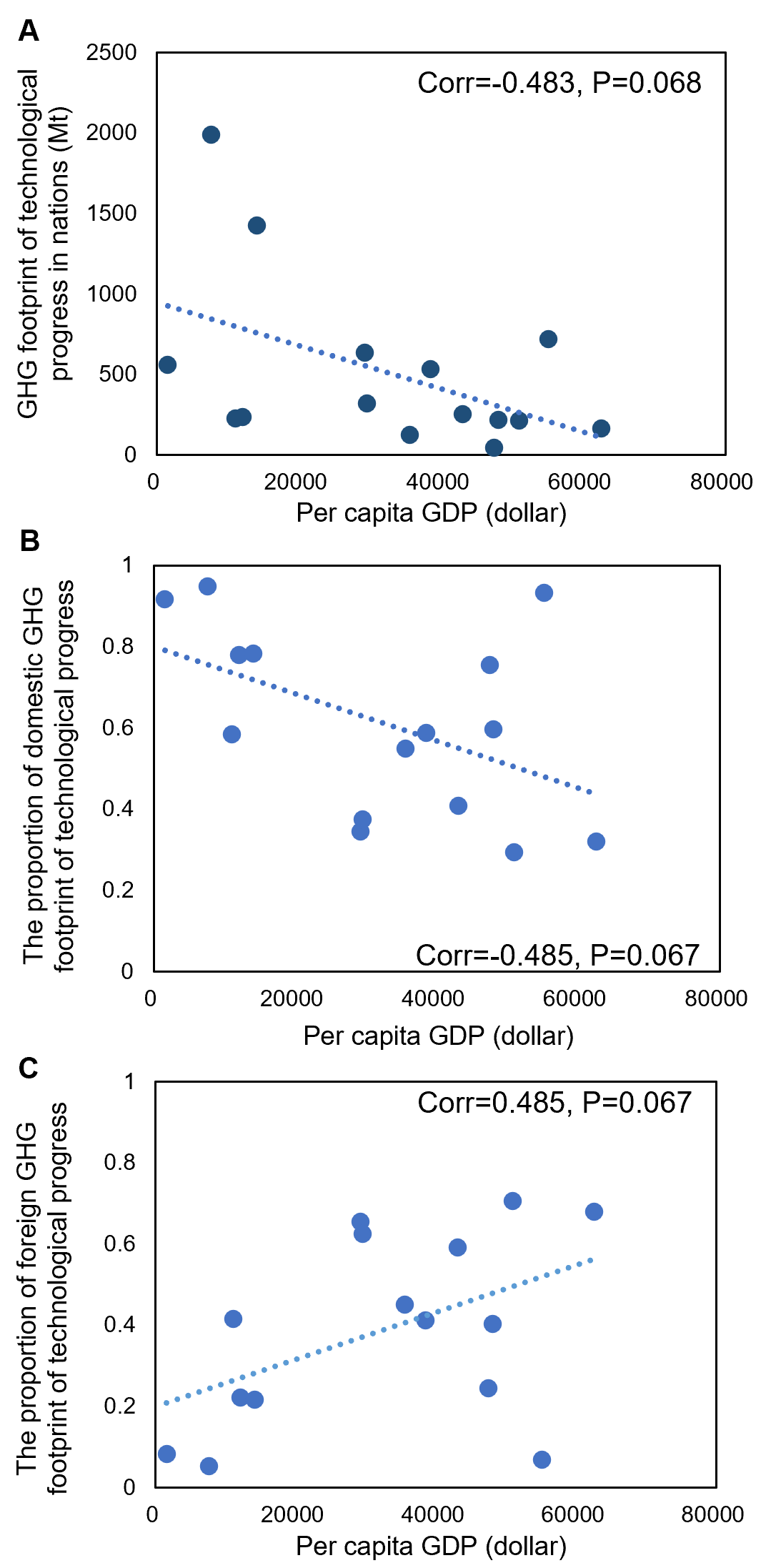


**Figure S3.** **Relationship between GHG footprint of technological progress and per capita GDP in nations.** A Relationship between GHG footprint of technological progress and per capita GDP in nations. B Relationship between the proportion of domestic GHG footprint of technological progress and per capita GDP in nations. C Relationship between the proportion of foreign GHG footprint of technological progress and per capita GDP in nations.





**Figure S4. Intersectoral linkages for the effects of technological progress (TP) in sectors of South Korea on greenhouse gas (GHG) emission increments of sectors in China.**


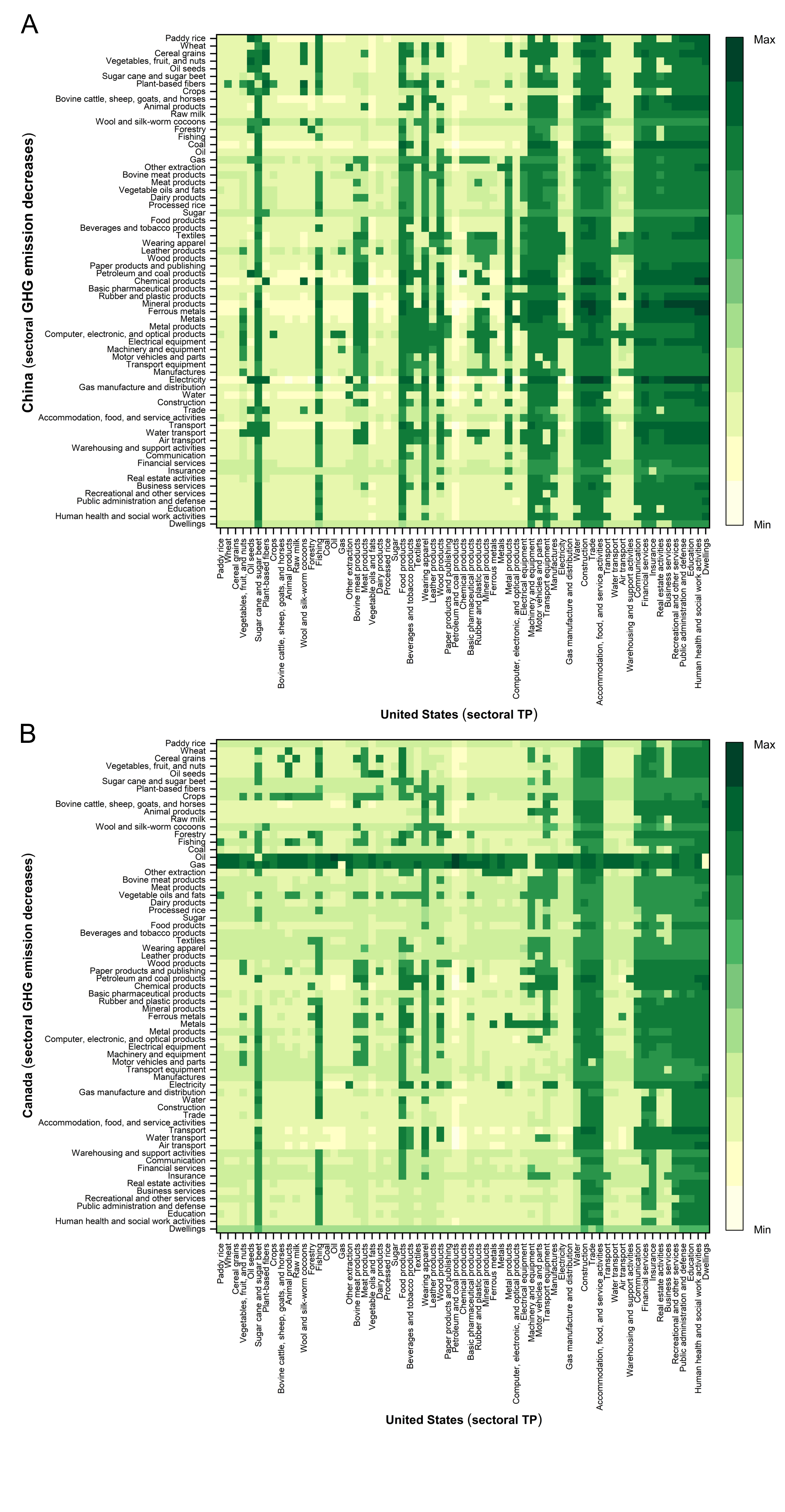


**Figure S5. Intersectoral linkages for the effects of technological progress (TP) in sectors of United States on greenhouse gas (GHG) emission decreases of sectors in China and Canada, respectively.**


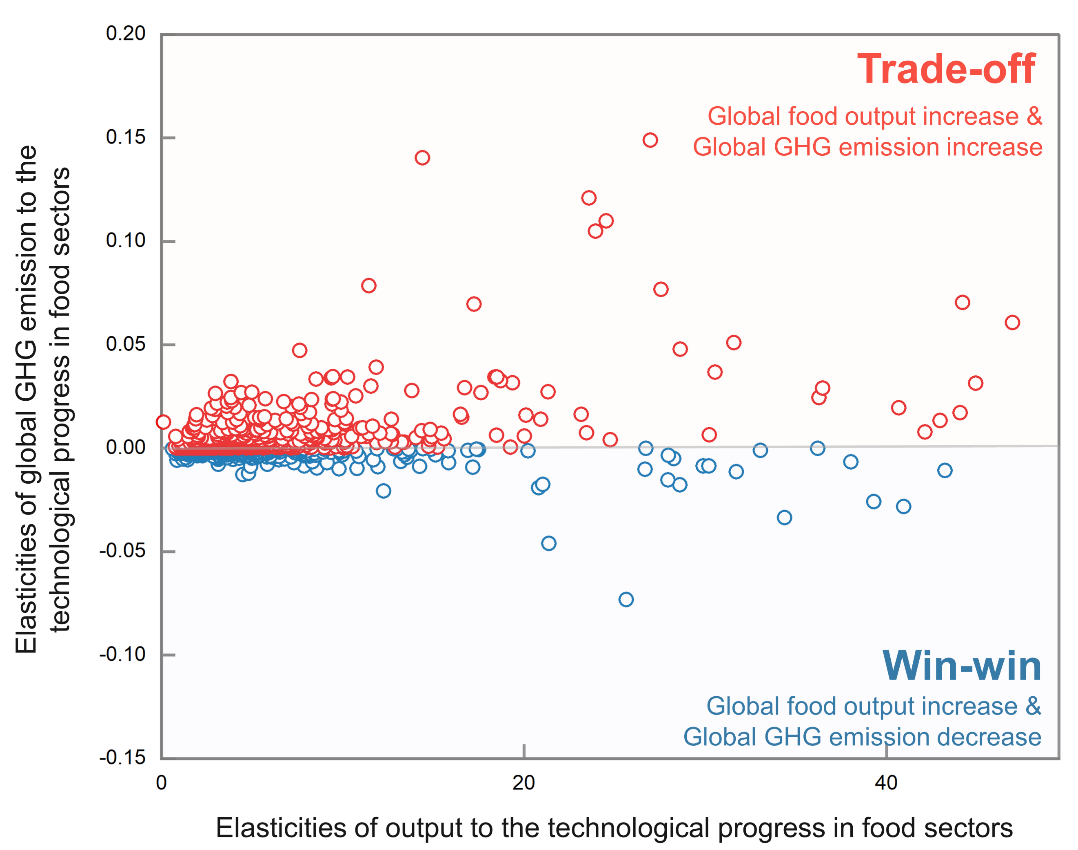


**Figure S6. Elasticities of food outputs and global GHG emissions to technological progress (TP) in food sectors.** The elasticity of food output to TP of a food sector means the ratio of changing rate of this food sector’s output to the changing rate of this sector’s technological level. The elasticity of global GHG emissions to TP of a food sector means the ratio of changing rate of global GHG emissions to the changing rate of this food sector’s technological level.

**Table S5. Greenhouse gas (GHG) emission categories and their data sources.**

| **Indexes** | **GHG** | **GHG emission categories** | | **Data sources** |
| --- | --- | --- | --- | --- |
| 1 | CO_2_ | Energy | Fossil fuel combustion activities | GTAP-E Data Base^1,2^ |
|  |  |  | Fugitive emissions from fuels: solid fuels | EDGAR (version 6.0) database^3^ |
|  |  |  | Fugitive emissions from fuels: oil and natural gas | EDGAR (version 6.0) database^3^ |
|  |  | Industrial processes and product use | Industrial processes and product use: cement production | EDGAR (version 6.0) database^3^ |
|  |  |  | Industrial processes and product use: lime production | EDGAR (version 6.0) database^3^ |
|  |  |  | Industrial processes and product use: glass production | EDGAR (version 6.0) database^3^ |
|  |  |  | Industrial processes and product use: chemical industry | EDGAR (version 6.0) database^3^ |
|  |  |  | Industrial processes and product use: metal industry | EDGAR (version 6.0) database^3^ |
|  |  | Wastes | Incineration and open burning of wastes | EDGAR (version 6.0) database^3^ |
| 2 | CH_4_ | Energy | Fossil fuel combustion activities | Non-CO_2_ Data Base^4^ |
|  |  |  | Fugitive emissions from fuels: solid fuels | Non-CO_2_ Data Base^4^ |
|  |  |  | Fugitive emissions from fuels: oil and natural gas | Non-CO_2_ Data Base^4^ |
|  |  | Industrial processes and Product use | Industrial processes and product use: chemical industry | Non-CO_2_ Data Base^4^ |
|  |  |  | Industrial processes and product use: metal industry | Non-CO_2_ Data Base^4^ |
|  |  | Agriculture | Enteric fermentation | Non-CO_2_ Data Base^4^ |
|  |  |  | Manure management | Non-CO_2_ Data Base^4^ |
|  |  |  | Emissions from biomass burning | Non-CO_2_ Data Base^4^ |
|  |  |  | Rice cultivations | Non-CO_2_ Data Base^4^ |
|  |  | Wastes | Solid wastes disposal | Non-CO_2_ Data Base^4^ |
|  |  |  | Biological treatment of solid wastes | Non-CO_2_ Data Base^4^ |
|  |  |  | Incineration and open burning of wastes | Non-CO_2_ Data Base^4^ |
|  |  |  | Wastewater treatment and discharge | Non-CO_2_ Data Base^4^ |
| 3 | N_2_O | Energy | Fossil fuel combustion activities | Non-CO_2_ Data Base^4^ |
|  |  |  | Fugitive emissions from fuels: oil and natural gas | Non-CO_2_ Data Base^4^ |
|  |  | Industrial processes and product use | Industrial processes and product use: chemical industry | Non-CO_2_ Data Base^4^ |
|  |  |  | Other product manufacture and use | Non-CO_2_ Data Base^4^ |
|  |  | Agriculture | Manure management | Non-CO_2_ Data Base^4^ |
|  |  |  | Emissions from biomass burning | Non-CO_2_ Data Base^4^ |
|  |  |  | Direct N_2_O emissions from managed soils | Non-CO_2_ Data Base^4^ |
|  |  |  | Indirect N_2_O emissions from managed soils | Non-CO_2_ Data Base^4^ |
|  |  |  | Indirect N_2_O Emissions from manure management | Non-CO_2_ Data Base^4^ |
|  |  | Wastes | Biological treatment of solid wastes | Non-CO_2_ Data Base^4^ |
|  |  |  | Incineration and open burning of wastes | Non-CO_2_ Data Base^4^ |
|  |  |  | Wastewater treatment and discharge | Non-CO_2_ Data Base^4^ |
| 4 | F-gas  (CF_4_, HFC134A, HFC23, SF_6_) | Industrial Processes and Product Use | Process emissions of primary aluminium production | Non-CO_2_ Data Base^4^ |
|  |  |  | Process emissions of SF_6_ used in aluminium production | Non-CO_2_ Data Base^4^ |
|  |  |  | Process emissions of SF_6_ used in magnesium foundries | Non-CO_2_ Data Base^4^ |
|  |  |  | Byproduct emissions of production of halocarbons and sulphur hexafluoride | Non-CO_2_ Data Base^4^ |
|  |  |  | Consumption of halocarbons and sulphur hexafluoride: refrigerator and air conditioning | Non-CO_2_ Data Base^4^ |
|  |  |  | Consumption of halocarbons and sulphur hexafluoride: foam blowing | Non-CO_2_ Data Base^4^ |
|  |  |  | Consumption of halocarbons and sulphur hexafluoride: fire extinguishers | Non-CO_2_ Data Base^4^ |
|  |  |  | Consumption of halocarbons and sulphur hexafluoride: aerosols | Non-CO_2_ Data Base^4^ |
|  |  |  | Consumption of halocarbons: semiconductors manufacturing | Non-CO_2_ Data Base^4^ |
|  |  |  | Consumption of halocarbons: flat panel display production | Non-CO_2_ Data Base^4^ |
|  |  |  | Consumption of halocarbons: photovoltaic cells manufacturing | Non-CO_2_ Data Base^4^ |
|  |  |  | Consumption of halocarbons: electrical equipment | Non-CO_2_ Data Base^4^ |
|  |  |  | Consumption of halocarbons: electrical equipment use | Non-CO_2_ Data Base^4^ |
|  |  |  | Other F-gas use and SF_6_ | Non-CO_2_ Data Base^4^ |
|  |  |  | Other F-gas use and SF_6_: adiabatic prop: shoes and others | Non-CO_2_ Data Base^4^ |
|  |  |  | Other F-gas use and SF_6_: adiabatic prop: tires | Non-CO_2_ Data Base^4^ |
|  |  |  | Other F-gas use and SF_6_: sound proof windows | Non-CO_2_ Data Base^4^ |
|  |  |  | Other F-gas use and SF_6_: accelerators | Non-CO_2_ Data Base^4^ |
|  |  |  | Other F-gas use and SF_6_: AWACS, other military, misc. | Non-CO_2_ Data Base^4^ |
|  |  |  | Other F-gas use and SF_6_: unknown SF_6_ use | Non-CO_2_ Data Base^4^ |

**Table S6. Mapping between CO_2_ emission categories of EDGAR database and GTAP sectors.**

| **CO_2_** **emission categories of EDGAR database** | | **GTAP sectors** | **Mapping methods** |
| --- | --- | --- | --- |
| Energy | Fugitive emissions from fuels: solid fuels | Coal^4,5^ | / |
|  | Fugitive emissions from fuels: oil and natural gas | Oil; Gas; Petroleum, coal products; and Gas manufacture, distribution^4,5^ | Assuming that sectors listed in the left cell have the same CO_2_ emission intensity, we allocate fugitive emissions from oil and natural gas to these sectors based on their total outputs from GTAP (version 10) database^1^. |
| Industrial processes and product use | Non-metallic mineral products (including cement production, lime production, and glass production) | Mineral products^4,5^ | / |
|  | Chemical Industry | Chemical products^4,5^ | / |
|  | Metal industry | Ferrous metals; and Other metals^4,5^ | Assuming that the two sectors listed in the left cell have the same CO_2_ emission intensity, we allocate CO_2_ emissions from the metal industry to these two sectors based on their total outputs from GTAP (version 10) database^1^. |
| Wastes | Incineration and open burning of wastes | Coal; Oil; Gas; Other extraction; Bovine meat products; Other meat products; Vegetable oils and fats; Dairy products; Processed rice; Sugar; Other food products; Beverages and tobacco products; Textiles; Wearing apparel; Leather products; Wood products; Paper products, publishing; Petroleum, coal products; Chemical products; Basic pharmaceutical products; Rubber and plastic products; Mineral products; Ferrous metals; Other metals; Metal products; Computer, electronic and optical products; Machinery and equipment; Motor vehicles and parts; Transport equipment; Other Manufactures; and Water^4-6^ | Assuming that sectors listed in the left cell have the same CO_2_ emission intensity, we allocate CO_2_ emissions from the incineration and open burning of wastes to these sectors based on their total outputs from GTAP (version 10) database^1^. |

**Table S8. Classification of sub-global areas in the world**

| **Indexes** | **Nations/Areas** | **Indexes** | **Sub-global areas** |
| --- | --- | --- | --- |
| 1 | Australia | R1 | Oceania |
| 2 | New Zealand |  |  |
| 3 | Rest of Oceania |  |  |
| 4 | China | R2 | East Asia |
| 5 | Hong Kong, China |  |  |
| 6 | Japan |  |  |
| 7 | South Korea |  |  |
| 8 | Mongolia |  |  |
| 9 | Taiwan, China |  |  |
| 10 | Rest of East Asia |  |  |
| 11 | Brunei Darussalam | R3 | Southeast Asia |
| 12 | Cambodia |  |  |
| 13 | Indonesia |  |  |
| 14 | Lao People's Democratic Republic |  |  |
| 15 | Malaysia |  |  |
| 16 | Philippines |  |  |
| 17 | Singapore |  |  |
| 18 | Thailand |  |  |
| 19 | Viet Nam |  |  |
| 20 | Rest of Southeast Asia |  |  |
| 21 | Bangladesh | R4 | South Asia |
| 22 | India |  |  |
| 23 | Nepal |  |  |
| 24 | Pakistan |  |  |
| 25 | Sri Lanka |  |  |
| 26 | Rest of South Asia |  |  |
| 27 | Canada | R5 | North America |
| 28 | United States |  |  |
| 29 | Mexico |  |  |
| 30 | Rest of North America |  |  |
| 31 | Argentina | R6 | Latin America and the Caribbean |
| 32 | Plurinational State of Bolivia |  |  |
| 33 | Brazil |  |  |
| 34 | Chile |  |  |
| 35 | Colombia |  |  |
| 36 | Ecuador |  |  |
| 37 | Paraguay |  |  |
| 38 | Peru |  |  |
| 39 | Uruguay |  |  |
| 40 | Venezuela |  |  |
| 41 | Rest of South America |  |  |
| 42 | Costa Rica |  |  |
| 43 | Guatemala |  |  |
| 44 | Honduras |  |  |
| 45 | Nicaragua |  |  |
| 46 | Panama |  |  |
| 47 | El Salvador |  |  |
| 48 | Rest of Central America |  |  |
| 49 | Dominican Republic |  |  |
| 50 | Jamaica |  |  |
| 51 | Puerto Rico |  |  |
| 52 | Trinidad and Tobago |  |  |
| 53 | Caribbean |  |  |
| 54 | Austria | R7 | Europe |
| 55 | Belgium |  |  |
| 56 | Bulgaria |  |  |
| 57 | Croatia |  |  |
| 58 | Cyprus |  |  |
| 59 | Czech Republic |  |  |
| 60 | Denmark |  |  |
| 61 | Estonia |  |  |
| 62 | Finland |  |  |
| 63 | France |  |  |
| 64 | Germany |  |  |
| 65 | Greece |  |  |
| 66 | Hungary |  |  |
| 67 | Ireland |  |  |
| 68 | Italy |  |  |
| 69 | Latvia |  |  |
| 70 | Lithuania |  |  |
| 71 | Luxembourg |  |  |
| 72 | Malta |  |  |
| 73 | Netherlands |  |  |
| 74 | Poland |  |  |
| 75 | Portugal |  |  |
| 76 | Romania |  |  |
| 77 | Slovakia |  |  |
| 78 | Slovenia |  |  |
| 79 | Spain |  |  |
| 80 | Sweden |  |  |
| 81 | United Kingdom |  |  |
| 82 | Switzerland |  |  |
| 83 | Norway |  |  |
| 84 | Rest of EFTA (European Free Trade Association) |  |  |
| 85 | Albania |  |  |
| 86 | Belarus |  |  |
| 87 | Russian |  |  |
| 88 | Ukraine |  |  |
| 89 | Rest of Eastern Europe |  |  |
| 90 | Rest of Europe |  |  |
| 91 | Kazakhstan | R8 | Central Asia |
| 92 | Kyrgyzstan |  |  |
| 93 | Tajikistan |  |  |
| 94 | Rest of Former Soviet Union |  |  |
| 95 | Armenia | R9 | Western Asia |
| 96 | Azerbaijan |  |  |
| 97 | Georgia |  |  |
| 98 | Bahrain |  |  |
| 99 | Islamic Republic of Iran |  |  |
| 100 | Israel |  |  |
| 101 | Jordan |  |  |
| 102 | Kuwait |  |  |
| 103 | Oman |  |  |
| 104 | Qatar |  |  |
| 105 | Saudi Arabia |  |  |
| 106 | Turkey |  |  |
| 107 | United Arab Emirates |  |  |
| 108 | Rest of Western Asia |  |  |
| 109 | Egypt | R10 | North Africa |
| 110 | Morocco |  |  |
| 111 | Tunisia |  |  |
| 112 | Rest of North Africa |  |  |
| 113 | Benin | R11 | Sub-Saharan Africa |
| 114 | Burkina Faso |  |  |
| 115 | Cameroon |  |  |
| 116 | Cote d'Ivoire |  |  |
| 117 | Ghana |  |  |
| 118 | Guinea |  |  |
| 119 | Nigeria |  |  |
| 120 | Senegal |  |  |
| 121 | Togo |  |  |
| 122 | Rest of Western Africa |  |  |
| 123 | Central Africa |  |  |
| 124 | South Central Africa |  |  |
| 125 | Ethiopia |  |  |
| 126 | Kenya |  |  |
| 127 | Madagascar |  |  |
| 128 | Malawi |  |  |
| 129 | Mauritius |  |  |
| 130 | Mozambique |  |  |
| 131 | Rwanda |  |  |
| 132 | United Republic of Tanzania |  |  |
| 133 | Uganda |  |  |
| 134 | Zambia |  |  |
| 135 | Zimbabwe |  |  |
| 136 | Rest of Eastern Africa |  |  |
| 137 | Botswana |  |  |
| 138 | Namibia |  |  |
| 139 | South Africa |  |  |
| 140 | Rest of South African Customs |  |  |
| 141 | Rest of the World | R12 | Rest of the World |

**Table S9.** **The names of sectors**

| **Indexes** | **Sector names in GTAP** | **Sector names in this study** |
| --- | --- | --- |
| 1 | Paddy rice | Paddy rice |
| 2 | Wheat | Wheat |
| 3 | Cereal grains nec | Cereal grains |
| 4 | Vegetables, fruit, nuts | Vegetables, fruit, and nuts |
| 5 | Oil seeds | Oil seeds |
| 6 | Sugar cane, sugar beet | Sugar cane and sugar beet |
| 7 | Plant-based fibers | Plant-based fibers |
| 8 | Crops nec | Crops |
| 9 | Bovine cattle, sheep and goats, horses | Bovine cattle, sheep, goats, and horses |
| 10 | Animal products nec | Animal products |
| 11 | Raw milk | Raw milk |
| 12 | Wool, silk-worm cocoons | Wool and silk-worm cocoons |
| 13 | Forestry | Forestry |
| 14 | Fishing | Fishing |
| 15 | Coal | Coal |
| 16 | Oil | Oil |
| 17 | Gas | Gas |
| 18 | Other Extraction (formerly omn Minerals nec) | Other extraction |
| 19 | Bovine meat products | Bovine meat products |
| 20 | Meat products nec | Meat products |
| 21 | Vegetable oils and fats | Vegetable oils and fats |
| 22 | Dairy products | Dairy products |
| 23 | Processed rice | Processed rice |
| 24 | Sugar | Sugar |
| 25 | Food products nec | Food products |
| 26 | Beverages and tobacco products | Beverages and tobacco products |
| 27 | Textiles | Textiles |
| 28 | Wearing apparel | Wearing apparel |
| 29 | Leather products | Leather products |
| 30 | Wood products | Wood products |
| 31 | Paper products, publishing | Paper products and publishing |
| 32 | Petroleum, coal products | Petroleum and coal products |
| 33 | Chemical products | Chemical products |
| 34 | Basic pharmaceutical products | Basic pharmaceutical products |
| 35 | Rubber and plastic products | Rubber and plastic products |
| 36 | Mineral products nec | Mineral products |
| 37 | Ferrous metals | Ferrous metals |
| 38 | Metals nec | Metals |
| 39 | Metal products | Metal products |
| 40 | Computer, electronic and optical products | Computer, electronic, and optical products |
| 41 | Electrical equipment | Electrical equipment |
| 42 | Machinery and equipment nec | Machinery and equipment |
| 43 | Motor vehicles and parts | Motor vehicles and parts |
| 44 | Transport equipment nec | Transport equipment |
| 45 | Manufactures nec | Manufactures |
| 46 | Electricity | Electricity |
| 47 | Gas manufacture, distribution | Gas manufacture and distribution |
| 48 | Water | Water |
| 49 | Construction | Construction |
| 50 | Trade | Trade |
| 51 | Accommodation, Food and service activities | Accommodation, food, and service activities |
| 52 | Transport nec | Transport |
| 53 | Water transport | Water transport |
| 54 | Air transport | Air transport |
| 55 | Warehousing and support activities | Warehousing and support activities |
| 56 | Communication | Communication |
| 57 | Financial services nec | Financial services |
| 58 | Insurance (formerly isr) | Insurance |
| 59 | Real estate activities | Real estate activities |
| 60 | Business services nec | Business services |
| 61 | Recreational and other services | Recreational and other services |
| 62 | Public Administration and defense | Public administration and defense |
| 63 | Education | Education |
| 64 | Human health and social work activities | Human health and social work activities |
| 65 | Dwellings | Dwellings |

**References**

1 Aguiar, A., Chepeliev, M., Corong, E., McDougall, R. & van der Mensbrugghe, D. The GTAP data base: Version 10. *Journal of Global Economic Analysis* **4**, 1-27 (2019).

2 Andrew, R. M. & Peters, G. P. A multi-region input–output table based on the global trade analysis project database (GTAP-MRIO). *Economic Systems Research* **25**, 99-121 (2013).

3 Crippa, M. *et al.* EDGAR v6.0 greenhouse gas emissions. (The European Union, 2021).

4 Chepeliev, M. Development of the non-CO_2_ GHG emissions database for the GTAP data base Version 10A (Indiana, 2020).

5 United Nations. International standard industrial classification of all economic activities. Revision 4 edition (Genève, 2008).

6 United Nations. Central product classification. Version 2.1 (New York, 2015).
